# On Holo-Hilbert spectral analysis: a full informational spectral representation for nonlinear and non-stationary data

**DOI:** 10.1098/rsta.2015.0206

**Published:** 2016-04-13

**Authors:** Norden E. Huang, Kun Hu, Albert C. C. Yang, Hsing-Chih Chang, Deng Jia, Wei-Kuang Liang, Jia Rong Yeh, Chu-Lan Kao, Chi-Hung Juan, Chung Kang Peng, Johanna H. Meijer, Yung-Hung Wang, Steven R. Long, Zhauhua Wu

**Affiliations:** 1 Research Center for Adaptive Data Analysis, National Central University, Zhongli 32001, Taiwan, Republic of China; 2 Graduate Institute of Cognitive Neuroscience, National Central University, Zhongli 32001, Taiwan, Republic of China; 3 Medical Biodynamics Program, Division of Sleep Medicine, Brigham and Women’s Hospital/Harvard Medical School, 221 Longwood Avenue, Boston, MA 02115, USA; 4 Department of Psychiatry, Taipei Veteran General Hospital, Shipai 11217, Taiwan, Republic of China; 5 The First Research Institution of Oceanography, SOA, Qingdao 266061, People’s Republic of China; 6 Beth Israel Deaconess Medical Center, Harvard Medical School, 330 Brookline Avenue, Boston, MA 02215, USA; 7 Department of Molecular Cell Biology, Laboratory for Neurophysiology, Leiden University Medical Center, 2300 RC Leiden, The Netherlands; 8 NASA GSFC, Sciences and Exploration Directorate, Field Support Office, Code 610.W, Wallops Flight Facility, Wallops Island, VA 23337, USA; 9 Department of Meteorology, Florida State University, 2035 E. Paul Dirac Drive, 200 R.M. Johnson Building, Tallahassee, FL 32306-2840, USA

**Keywords:** Hilbert–Huang transform, nonlinear, non-stationary, empirical mode decomposition, Holo-Hilbert spectrum, Holo-Hilbert spectral analysis

## Abstract

The Holo-Hilbert spectral analysis (HHSA) method is introduced to cure the deficiencies of traditional spectral analysis and to give a full informational representation of nonlinear and non-stationary data. It uses a nested empirical mode decomposition and Hilbert–Huang transform (HHT) approach to identify intrinsic amplitude and frequency modulations often present in nonlinear systems. Comparisons are first made with traditional spectrum analysis, which usually achieved its results through convolutional integral transforms based on additive expansions of an *a priori* determined basis, mostly under linear and stationary assumptions. Thus, for non-stationary processes, the best one could do historically was to use the time–frequency representations, in which the amplitude (or energy density) variation is still represented in terms of time. For nonlinear processes, the data can have both amplitude and frequency modulations (intra-mode and inter-mode) generated by two different mechanisms: linear additive or nonlinear multiplicative processes. As all existing spectral analysis methods are based on additive expansions, either *a priori* or adaptive, none of them could possibly represent the multiplicative processes. While the earlier adaptive HHT spectral analysis approach could accommodate the intra-wave nonlinearity quite remarkably, it remained that any inter-wave nonlinear multiplicative mechanisms that include cross-scale coupling and phase-lock modulations were left untreated. To resolve the multiplicative processes issue, additional dimensions in the spectrum result are needed to account for the variations in both the amplitude and frequency modulations simultaneously. HHSA accommodates all the processes: additive and multiplicative, intra-mode and inter-mode, stationary and non-stationary, linear and nonlinear interactions. The *Holo* prefix in HHSA denotes a multiple dimensional representation with both additive and multiplicative capabilities.

## 1. Introduction

In scientific and engineering studies, spectral analysis is a powerful tool to reveal the statistical characteristics of stochastic data. The spectral analysis method can be viewed as any operation to transform temporal data of arbitrary length into a frequency representation of finite domain between 1/*T*, with *T* as the total data length, and the Nyquist frequency, *f*_N_, given by
1.1
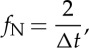
with Δ*t* as the sampling rate. This time-to-frequency conversion would make it much easier to assess the statistical properties of the data in a fixed and finite frequency range. Indeed, it has become the standard tool in studying all kinds of stochastic phenomena, from ocean waves, turbulence, earthquake, speech, structure and machine vibrations to biomedical research as in electroencephalogram analysis and heart rate variability.

As powerful as the traditional spectral analysis has been, all the available methods are based on additive expansions. Taking Fourier analysis as an example, we have
1.2
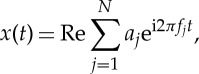
in which Re is for the real part of the expansion. Obviously, for the additive expansions with a constant amplitude, *a*_*j*_, and a constant frequency, *f*_*j*_, the Fourier-based spectral analysis can only have physical meaning for linear stationary processes.

Recent advances in wavelet [1] and Hilbert spectral analyses (HSAs) in [2,3] have added the temporal variations of the frequency in the form of a time–frequency representation. Two novel ideas were introduced by the HSA. First, the whole expansion is based on an adaptive intrinsic mode function (IMF) basis obtained through empirical mode decomposition (EMD), given as
1.3
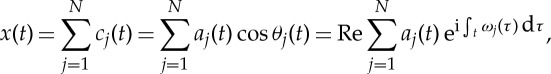
where the frequency is defined as the time derivative of the adaptively determined phase function, *θ*_*j*_(*t*). The result is no longer a mean value over the time domain determined by any of the integral transforms, but a result that now has instantaneous values at different times. Secondly, this adaptive expansion also enables us to represent both the frequency and amplitude *a*_*j*_(*t*) as functions of time. Thus, spectral analysis can reveal time-dependent variations, a standard method for non-stationary processes. In particular, the intra-wave frequency variations can yield a measure of the degree of nonlinearity within each IMF mode [4].

Even with these generalizations, the existing spectral analysis methods still contain serious limitations. Essentially, all the above spectral analyses are based on additive expansions, and the frequency variations are determined by the fast-changing carrier waves, *ω*(*τ*). Through this approach, the time variations of *a*(*t*) and *ω*(*τ*) would cover the non-stationary processes, but the amplitude (or the energy proportional to the square of amplitude) variations would still be in terms of time functions. This carrier frequency-based approach made the HSA extremely effective and powerful in intra-mode frequency-modulated (FM) signals; however, it has neglected the role of the inter-mode amplitude and frequency modulations (AMs and FMs). What are the significant aspects of amplitude modulation?

In the initial physical measurements, there are two ways the amplitude of a signal from a complex system could be modulated: by additions or by multiplications among the participating components, as in [5]. A distinct advantage for decomposing the time series into IMFs is that all the additive interactions can be separated, extracted and quantified by the EMD and HSA, as illustrated in numerous situations discussed in [2,6]. Although EMD is still linear in form, its result can have nonlinear properties that produce IMFs with amplitude and frequency variations. However, those earlier works left the AMs untreated, which are the results of the inter-scale and cross-scale interactions. These also signify the phase-locked modulations. All these phenomena are the consequences of truly nonlinear processes. Historically, the Fourier spectral analysis approach has ignored this AM by assuming the amplitude to be constant, an assumption that is totally unjustifiable. HSA treated the amplitude as a temporal fluctuation that resulted in a mixed time–frequency representation; any such representations should be viewed as incomplete spectral transforms.

As most of the natural or man-made signals contain both AM and FM characteristics, it behoves us to ask: What information have we missed in the existing methods of spectral analysis? What are the implications and the physical significance of AM variations? Finally, if the amplitude is indeed critical, then how can we best represent the AM variations in complete, full informational spectral representations?

In this paper, we propose a new Holo-Hilbert spectrum (HHS) which will answer these questions with a totally different approach: to establish AMs and FMs through higher-dimensional representations. In addition to the intra-mode approach in the HSA reported by Huang *et al.* [2,7], this new approach will enable us to examine the complicated inter-mode modulations explicitly and quantitatively. Additionally, the possibility of having FM expansions is also addressed. Thus, we would finally have a complete and full informational, high-dimensional view of any data from nonlinear and non-stationary processes. We would thus be able to examine the AM and FM variations simultaneously. This paper will include the following sections: §2 is on the mechanism of modulations; §3 will introduce the new full informational, complete frequency-domain representation, i.e. the Holo-Hilbert spectral analysis (HHSA) [8]; applications in practical examples will then be given in §4; §5 gives the definition, separation, extraction and quantification of time-dependent amplitude functions from a given dataset; and finally, there will be a section (§6) on discussion and conclusions.

## 2. The mechanism of linear and nonlinear modulations

**Figure 1.**
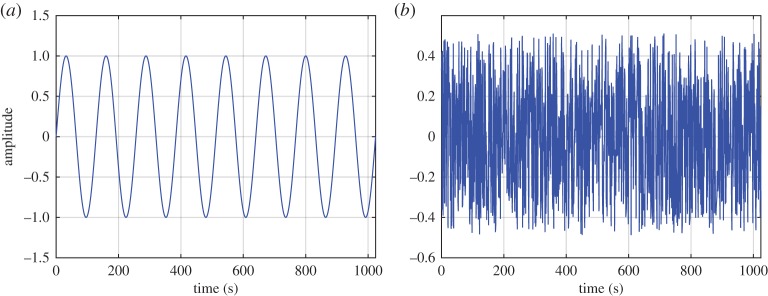
(*a*) The model sine wave. (*b*) The model Gaussian white noise signal. (Online version in colour.)

**Figure 2.**
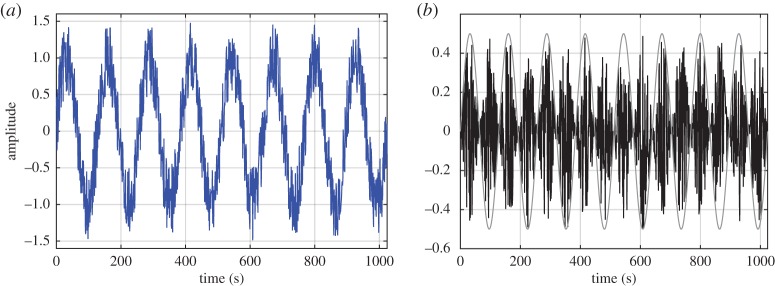
(*a*) Data for additive process: the sum of the sine wave and the white noise. (*b*) Data for multiplicative process: the product of the sine wave (thick grey line shown in background for comparison) and the white noise. (Online version in colour.)

**Figure 3.**
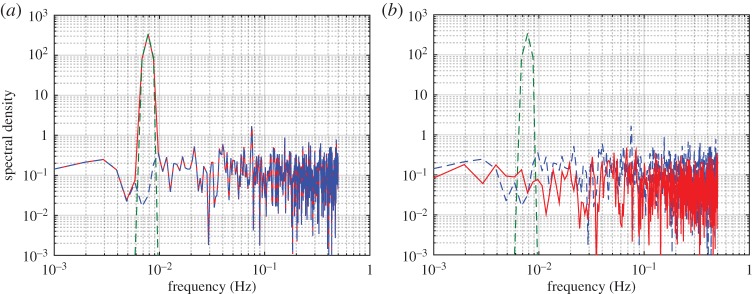
(*a*) The Fourier spectrum of the additive data: a simple superposition of the individual spectra from the sine and the white noise (the green dashed line for the sine wave, blue dotted line for noise and red solid line the sum). (*b*) The Fourier spectrum of the multiplicative data: the original sine wave disappeared; the overall product energy density actually decreases (the green dashed line for the sine wave, blue dotted line for noise and red solid line for the product; the energy density will increase if the sine wave amplitude is much larger than 1).

Most natural systems are inherently complex. Seldom would a signal be generated by an isolated force from a single source without interacting with other coexisting ambient variations. This is true especially for complicated living systems, in which forces of different scales are intertwined, and they interact both linearly (additively) and nonlinearly (multiplicatively). The fatal flaw of all the additive expansions is to reduce all multiplicative processes to additive ones. To simplify the discussion, let us consider the idealized case of dynamical interactions between a monochromatic wave and turbulence, represented by a pure sinusoidal wave and Gaussian distributed white noise (generated by the standard MatLab code) here, as given in figure 1*a*,*b*. If they are considered to be the velocity field of a moving fluid medium, then the total velocity field is given by
2.1
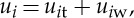
where the subscript ‘t’ stands for turbulence and ‘w’ for wave contributions. For simple incompressible fluids, the control equations for mass and momentum conservation should be
2.2
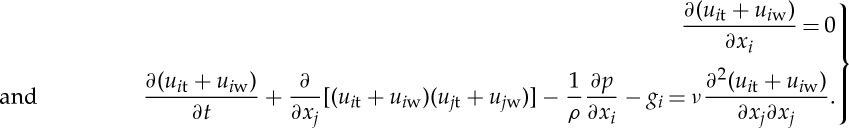
Here we can see that the kinematics of the field is given by the first expression of equations (2.2), while the dynamics is given by the second expression. Thus, the kinematics has linear (additive) properties, but the dynamics has nonlinear (multiplicative) interactions. Now let us examine the modulation mechanisms of the different interaction processes. The results of the additive and multiplicative interactions are illustrated in figure 2*a*,*b*. Clearly, we can see the effects of the wave oscillation in both datasets, giving large AMs. Let us now examine the data through the Fourier spectra summarized in figure 3*a*,*b*. The deficiency of the strictly linear representation in the Fourier analysis can be clearly seen. Figure 3*a* shows that the spectrum for data from the linear additive process is simply the sum of the spectra from the sinusoidal wave and the white noise. However, in figure 3*b* for data from the nonlinear multiplicative process, the trace of the sinusoidal wave is nowhere to be seen in the resulting spectrum, even though we can see the AMs of the sine wave on the white noise in the data clearly.

This seemingly puzzling result is actually very reasonable. Consider the case of two sinusoidal terms,
2.3
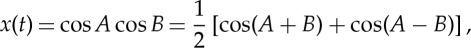
which could well be the case of a sinusoidal wave, cos *B*, with the AM by cos *A*. The Fourier spectral expansion will rely on a trigonometric identity to force the product term into the sum form. Mathematically, they are equivalent, but physically they are completely different: a single term, cos *A* times cos *B*, is changed into the sum of two waves simply because Fourier cannot represent a multiplicative operation.

The two-term case is somewhat ambiguous and not easy to see. This additive–multiplicative ambiguity is hard to resolve until we consider a case with three or more wave multiplications, for example:
2.4
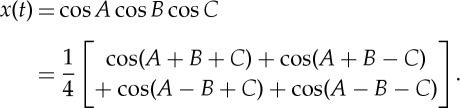
In fact, it can be shown that *n* product terms will end with the sum of 2^(*n*−1)^ waves. All the additive terms will congregate near the highest frequency component. This shifting of energy density to high frequency not only is uninformative, but also makes the spectral energy density inextricably mixed with noise and harmonics. To carry this argument a step further mathematically, let us consider the case of the sum and product of a sinusoidal modulating the white noise given above as
2.5
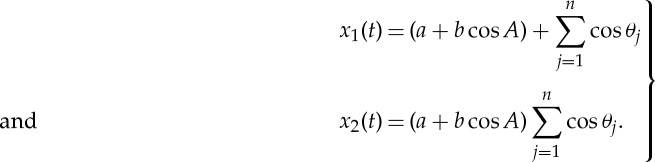
Without loss of generality, let us take *a*=0 and *b*=1. Then we would have the idealized case set up above, and
2.6
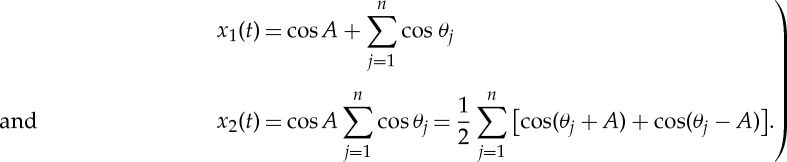
The values of functions *x*_1_(*t*) and *x*_2_(*t*) are exactly as given in figure 2*a*,*b*, with their respective Fourier spectra given in figure 3*a*,*b*.

The Fourier spectrum does not retain any trace of the clearly dominant modulating sinusoidal wave, because its energy has been evenly spread throughout the frequency range according to the second expression in equation (2.6). No amount of bandpass filtering could recover the lost signature of the modulating wave. As the energy of the sine wave has already been spread, the bandpassed results retain a very low energy level at the frequency of the sinusoidal wave. Furthermore, the frequency is a sum of the wave and the white noise, so there is no frequency-domain difference between the filtered results from the pure white noise and the multiplicative data, which is only a constant shift of the white noise. The signature of the all-important modulating sinusoidal wave that is clearly visible in raw data is lost totally by not considering the multiplicative processes. For these examples, the HSA will yield similar results as in the additive Fourier analysis, as will be shown later.

In a real dynamical setting such as with ocean surface waves, the velocity field should no longer be simply a superposition as given in equation (2.6). Instead, the dynamically modulated velocity will be moved by the background wave orbital velocity convectively. The detailed dynamics of the wave–turbulence interactions will be discussed by Qiao *et al.* [9] in this theme issue. Suffice it to say that the above results reveal a critical deficiency of the existing additive expansion-based spectral analysis methods in Fourier, wavelet and even HSA: the inability to represent multiplicative interactions. This is a fatal flaw. To quantify the multiplicative processes, we have to consider the AMs.

AMs, after the additive EMD, are produced by multiplicative processes. Multiplying two signals will produce AMs that would not be amenable to any additive decomposition methods. Multiplication is a nonlinear operation; it signifies cross-scale couplings and phase-locked modulations, which are hallmarks for nonlinear inter-mode processes. There are actually two distinct types of nonlinear effects: the intra- and inter-mode nonlinear phenomena. In each IMF mode, the control mechanisms could generate nonlinear waveform distortions as shown in all the classical nonlinear models from the logistic function to the Lorentz, Duffing and Rösseler equations [10], which are all the consequence of multiplicative (or higher-power) terms in the equations. But this multiplicative result could have the same time scale and reside in a single IMF. We designate this nonlinearity as intra-mode nonlinearity. The intra-mode nonlinear consequences reside in one IMF term as discussed in great detail by Huang *et al.* [4]. The inter-mode nonlinear effects are generated by cross-scale multiplicative processes. The dynamical consequences could be cross-scale coupling, which are phase-locked modulations that involve more than one IMF mode. Other than the single-mode phenomena studied by Huang *et al.* [2,4], the inability to quantify these inter-mode multiplicative nonlinear effects renders the existing spectral analysis of limited use in studying complex nonlinear systems. This is because the cross-scale interactions are of critical importance in such dynamical processes, even though the participating components in the multiplications could be linear signals. To explore the inter-mode nonlinear interactions, we have to find a new method to accentuate the multiplicative AMs.

It should be pointed out here that not all of the additive decomposition methods are equal. Fourier analysis is linear additive; it has no difficulty in extracting the additive information for data from linear and stationary processes. EMD [2] is also an additive decomposition method; it could extract additive AMs even for non-stationary and intra-mode nonlinear processes. None of the existing methods could extract multiplicative signals, signified by envelope modulations. For Fourier analysis, the amplitude values for each component are assigned to be constant, hence there is no way to examine the AM any more in that case. For EMD, however, any multiplicative process-induced energy intensity fluctuation could only be examined indirectly from the AM, which leads to the new high-dimensional, full informational spectral analysis to be given in the next section.

## 3. A full informational, complete frequency-domain representation:Holo-Hilbert spectral analysis

For any data, after the necessary first additive decomposition, we have the expansion given in equation (1.3). The carrier FM part has been covered in the HSA [7], in which the variation of the amplitude is implicitly expressed as the temporal modulations of the spectral intensity without further expansion or conversion into the frequency domain. Thus, this time–frequency analysis is in fact an incomplete spectral analysis.

**Figure 4.**
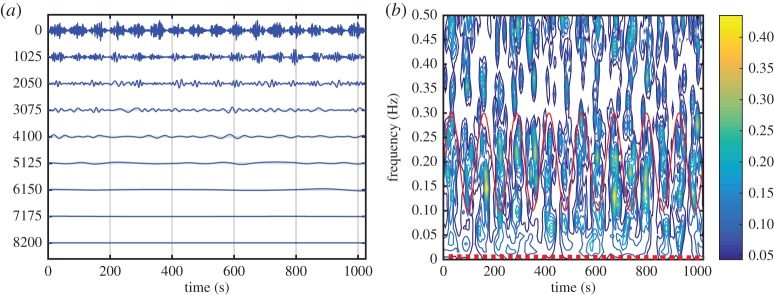
(*a*) The IMFs of the multiplicative data: the modulation by the sine wave can be seen in the almost regular amplitude variations in the first few IMFs (from the top down). (*b*) The Hilbert time–frequency spectrum of the multiplicative data: the modulation of the wave can be seen at the carrier frequency of the IMF with the modulating sine wave given by the red solid line. There is no trace of the sine wave at its frequency marked by the red dotted line.

Here, we are going to concentrate on the variation of the amplitude function, *a*_*j*_(*t*). In the above example of multiplicative interaction between the sinusoidal wave and the white noise, the IMFs of the data (in figure 2*b*) are given in figure 4*a* and the corresponding Hilbert spectrum is given in figure 4*b*. Although one can clearly see modulation of the IMFs especially in the first few IMF components, the Hilbert spectrum fails to reveal any trace of the modulation effects by the sinusoidal wave, which should appear along the dotted line given in this figure. The modulation can be seen only in the energy density fluctuation at the carrier frequency range. Neither Fourier nor the HSA could represent the effects of multiplicative processes, being as they are based on additive expansions (see §1).

Now let us examine the IMFs given in figure 4*a* again. Let us compute the envelopes for the IMFs with the steps as defined by Huang *et al.* [3,11]:

1. Take the absolute value of the IMFs.
2. Identify all the maxima of the absolute-valued function of IMFs.
3. Construct the envelopes by a natural spline through all the maxima.

**Figure 5.**
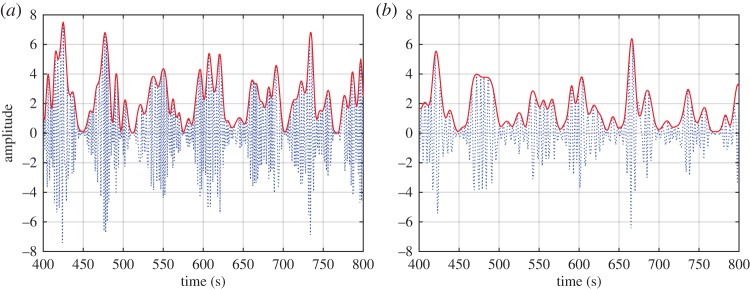
(*a*) Details of the AM of the first IMF component as marked by its envelope (the thick solid red line covering the higher frequency data), which has the correct frequency of the modulating sine wave. (*b*) Details of the AM of the second IMF component as marked by its envelope (the thick solid red line covering the higher frequency data), which has the correct frequency of the modulating sine wave. (Online version in colour.)

**Figure 6.**
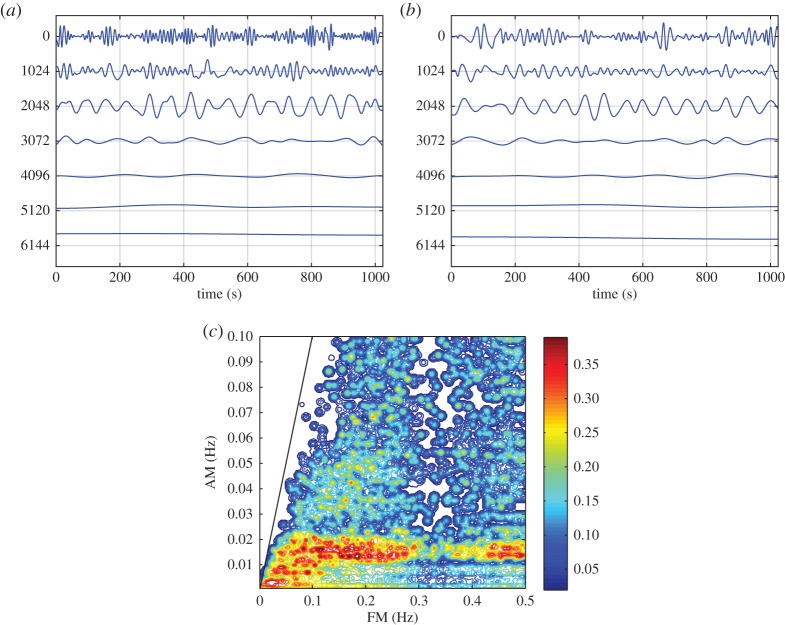
(*a*) The IMFs for the amplitude function given in figure 5*a*. The pattern of the modulating sine wave can be seen in the third IMF (counting from the top) by considering the horizontal scale of each oscillation. The vertical scale carries the units of the data, but in this plot, it is the horizontal scales that are emphasized to show the separation achieved. (*b*) The IMFs for the amplitude function given in figure 5*b*. Again, the pattern of the modulating sine wave can be seen in the third IMF (counting from the top) by considering the horizontal scale of each oscillation. The vertical scale carries the units of the data, but in this plot, it is the horizontal scales that are emphasized to show the separation achieved. (*c*) The HHS for the multiplicative data. The *x*-axis is the carrier wave frequency; the *y*-axis is the AM frequency. The contour represents the energy density. The prominent modulating sine wave frequency with high energy density can be clearly seen here. Because of the full modulation, this frequency is twice the value of the sine wave. The solid line indicates the boundary condition *ω*>*Ω*.

For the first few IMFs, the time fluctuations of the amplitude function clearly appear again. These envelopes are all positive-valued functions, representing the effects of the nonlinear multiplicative processes as shown in figure 5*a*,*b* as the lower frequency envelope compared with the higher carrier frequency oscillations. EMD analysis of the envelope functions produces the IMFs that contain the original signal of the modulating sine wave in component number 3 (third from the top) in both figure 6*a*,*b*. Perturbated by the coexisting noise, the recovered wave is not exactly a pure sine wave as seen in the C3; however, its wave structure indeed has the same mean frequency of the modulating waves to be shown presently.

**Figure 7.**
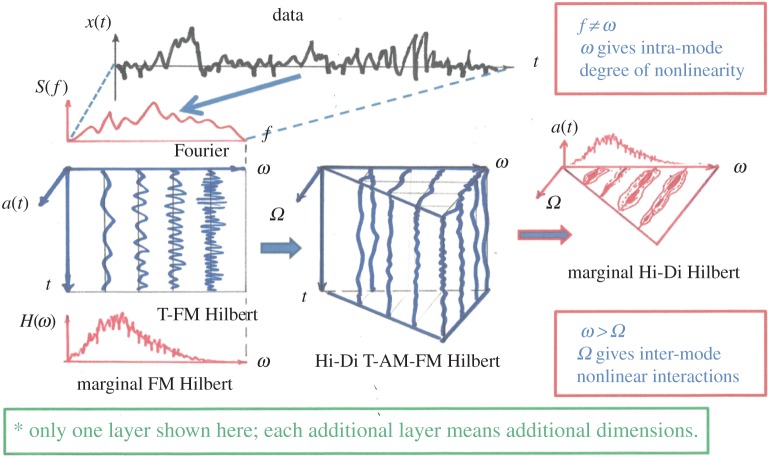
A schematic of the HHSA: the high-dimensional spectrum enables the full informational representation.

The procedures outlined above should be iterated to delineate the additive or multiplicative processes clearly in the data. Indeed, the modulations could be embedded in the data in many layers. Mathematically, the data analysis procedures are summarized as follows:
3.1
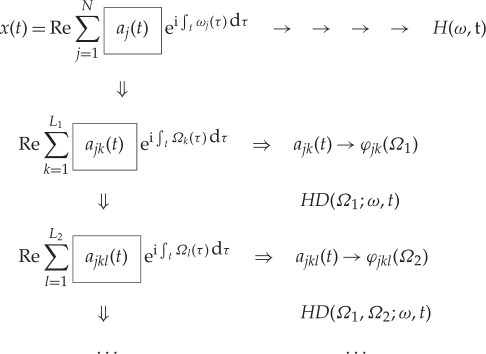
Therefore, to the second and higher layers, we essentially have the nested expression for the amplitude functions
3.2
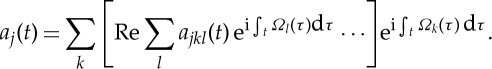
With these expressions, we have finished the expansion of data into the AM variations, covering fully multiplicative processes. The procedures to compute the original Hilbert time–frequency spectrum have been explained in great detail by Huang *et al.* [7]. Now, with each additional layer of decomposition on the amplitude envelope functions, *a*_*jk*_(*t*), *a*_*jkl*_(*t*), …, we have to add additional dimensions to accommodate the AM frequencies, *Ω*_1_, *Ω*_2_, …, to resolve the phase-AMs or inter-scale coupling effects. Consequently, the spectrum would become a genuine high-dimensional representation (*HH*_*k*_(*Ω*_1_, *Ω*_2_,…,*Ω*_*k*_; *ω*, *t*) in the schematic). At each step, the time variable can be integrated out to give a frequency-only spectrum (*hh*_*k*_(*Ω*_1_,*Ω*_2_,…,*Ω*_*k*_; *ω*) in the schematic, figure 7), but this is only a marginal expression. The procedure can go through as many iterations as needed until the amplitude functions bear no more cyclic characteristics in the envelopes. There would then be no further need for the time axis (no further variations in time), unless there is a monotonic trend, which could be removed easily as shown by Wu *et al.* [12]. When we reach this stage, we would have a pure multi-dimensional frequency representation of the data. The end result of this analysis is to expand the original time–frequency Hilbert spectrum to a higher-dimensional representation of the amplitude (AM)–frequency spectrum, with the frequency, *ω*, representing the fast-changing carrier intra-mode frequency variations and the AM frequency, *Ω*_*j*_, representing the slow-changing envelope inter-mode frequency variations, all related to energy density. By the dyadic nature of the IMFs, they are essentially zero-mean and narrow band except for the final trend. At each layer, the envelope is smoother than the carrier. Therefore, a finite number of iterations would reduce the final spectrum to a pure-frequency high-dimensional full informational representation. This new higher-dimensional spectral form is designated as the Holo-Hilbert AM spectrum (*HH*_*m*_(*Ω*_1_, *Ω*_2_,…,*Ω*_*m*_, *ω*) in the schematic). With this new full informational spectral representation, the signature of the sine wave can be easily identified in the HHS, as given in figure 6*c*. Here the modulation wave frequency is clearly shown, which is totally absent from the time–frequency representation given in figure 4*b*.

Thus, we have fully exploited the AM. Left untreated are the frequency variations and modulations. By analogy, we should also have the FM as
3.3
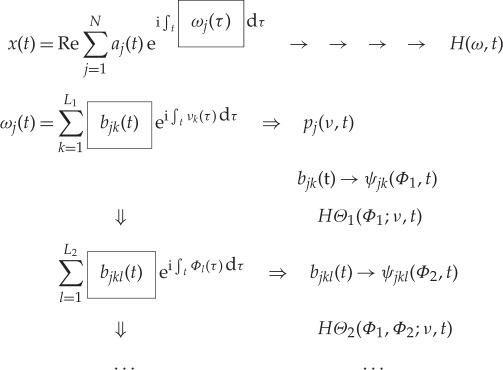
or
3.4
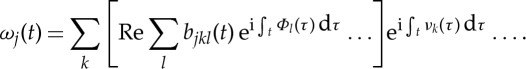
Now, we also have the FM counterpart of the Holo-Hilbert FM spectrum for the frequency. As the magnitude of the frequency variation is also unrelated to energy fluctuation, it is, therefore, less significant as far as energy density is concerned. However, it is essential for representing a chirp type of frequency when there are genuine FMs. Furthermore, it also represents the intra-mode degree of nonlinearity [4].

A special significance can also be assigned to the variation of the FM modulations, which could be extremely useful in revealing the amplitude–phase interactions. This variation can be constructed easily by combining the Hilbert energy spectrum, *H*(*ω*,*t*), with the sum of the first layer of frequency variations, *p*_*j*_(*ν*,*t*), to get
3.5
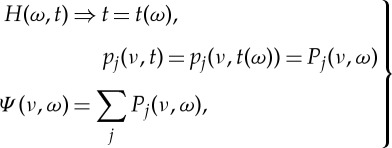
which actually relates the frequency variation of energy with the frequency variation in carrier wave instantaneous frequency. This special spectrum does not involve the envelopes of either amplitude or frequency; however, it reveals the instantaneous frequency variation with energy change, which is useful in determining the order of the intra-wave degree of nonlinearity [4]. This special spectrum is designated as the AM–FM spectrum. The AM–FM spectrum would be particularly effective in detecting the gradual frequency shift such as in a chirp signal.

The end result of this analysis is to expand the original time–frequency–energy Hilbert spectrum into a higher-dimensional representation of the multi-dimensional (or multi-layer) frequency (carrier)–frequency (AM) energy spectrum, with the FM frequency, *ω*, representing the fast-changing carrier intra-mode carrier frequency variations and the AM frequency, *Ω*_*j*_, representing the slow-changing inter-mode frequency variations. Schematically, the operations could be presented graphically as given in figure 7. Here, we present only one layer of the iterations. The traditional Fourier spectral analysis is a complete transform from time to frequency domain; however, its validity depends on stationary and linear assumptions. The Hilbert time–frequency spectrum extends the frequency into the time domain, but failed to provide the frequency-domain information on the amplitude function. Under stationary conditions, the Hilbert time–frequency spectrum could be summed into a marginal frequency–energy spectrum as the counterpart of the Fourier spectrum, but the meanings of the frequency are totally different [7], for the Hilbert spectrum is based on the physically meaningful instantaneous frequency [3].

The high-dimensional spectral representation would only fill half of the *ω*–*Ω* space, for *Ω* is derived from the slowly varying amplitude; therefore, the condition *ω*>*Ω* is always valid. As the spectrum becomes high-dimensional, visualization and graphical presentations would be a problem. In most of the cases reported here, the results are presented as a slice or a projection of the high-dimensional full spectrum.

The major expansion of the earlier time–frequency HSA proposed here is the transform of the amplitude function into the frequency domain, and then obtaining the high-dimensional AM and FM spectrum, HHS. As an example for simple processes, we have to go to only one additional layer. This new expansion would call for one additional dimension of frequency, *Ω*_1_. This four-dimensional spectrum is the complete full time–frequency spectral representation. For stationary processes, the time variation would carry no information, thus allowing a reduction of the four-dimensional spectrum into a three-dimensional spectrum. Alternatively, with multiple iterations, all temporal variations will eventually be extracted, for the envelope of an envelope is a fast smoothing operation. Furthermore, IMFs are all zero-mean and narrow band, so they thus enjoy some degree of stationarity in most cases. Therefore, a three-dimensional presentation would be sufficient to reveal a wealth of information to us in most cases.

A couple of key novel features should be stressed here. Although the Hilbert spectral approach could represent the nonlinear effects within one single IMF (the intra-mode nonlinearity), the new HHS would also include inter-mode nonlinear processes. All the traditional spectral representations, either Fourier or the Hilbert marginal spectra, are incomplete, for they neglect the nonlinear inter-mode interactions. It should be pointed out here again that all the additive interactions could be resolved by EMD. What the EMD failed to do was find the nonlinear inter-mode multiplicative interactions. Secondly, the schematic here represents only one layer of the expansions given in equation (3.3). The amplitude of each of the four-dimensional AM–FM spectra could be expanded further as explained above.

From the above discussions, we can see that the high-dimensional spectrum, HHS, actually represents the multiplicative interactions; it enables us to examine the details of all the nonlinear interactions either intra- or inter-mode. The inter-mode interactions are actually the result of the all-important cross-scale couplings or simply a coupled system [10], which were all left out in the past methods of spectral analysis.

## 4. Examples of Holo-Hilbert spectral analysis

**Figure 8.**
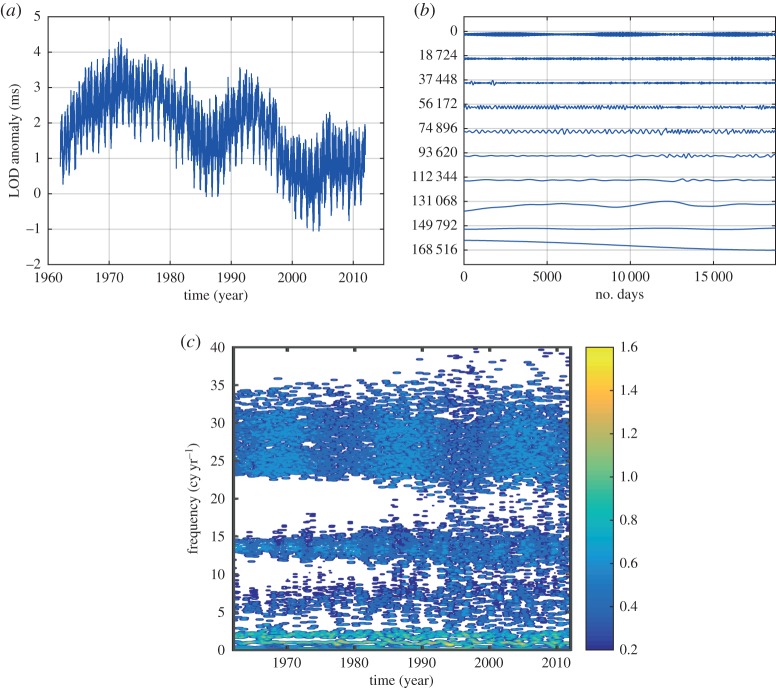
(*a*) The daily length-of-day (LOD) data from January 1962 to December 2012. (See Data accessibility section). (*b*) The IMFs from the LOD data: note the AM of the first few IMFs. The figures are produced by MatLab code (*strips*(*c*)): The *x*-axis is the number of data points; the *y*-axis is with arbitrary unit and the numbers again are the number of consecutive data points. (*c*) The Hilbert time–frequency representation of the LOD data: note the clear AMs of the half-monthly and monthly frequency bands of the Metonic (19 year) and Olympiad (4 year) cycles. Yet there is no energy at those frequency bands in this spectral representation.

**Figure 9.**
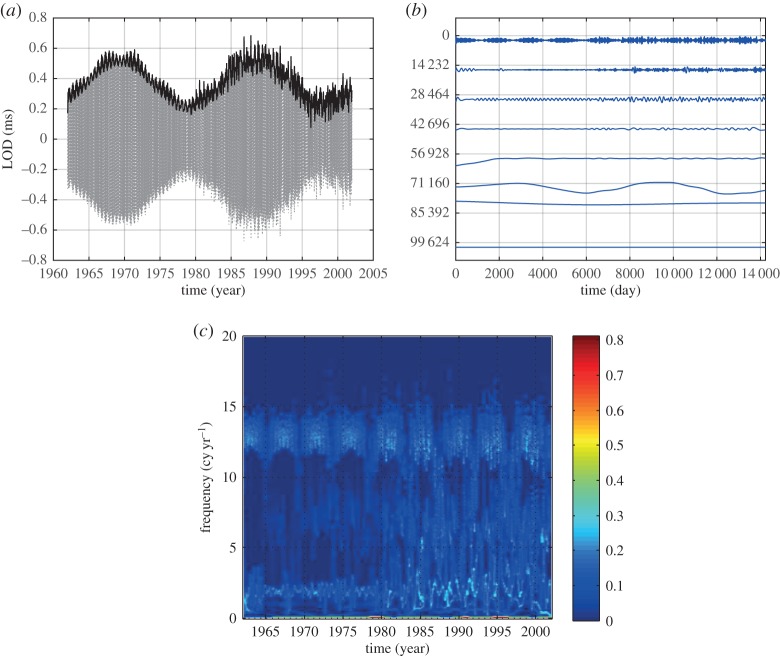
(*a*) The details of the half-monthly component in figure 8*b* and its amplitude envelope function (solid black line) showing the modulating pattern of the Metonic cycle (19 years). (*b*) The IMFs of the amplitude function given in figure 9*a*. Here we can see the clear AM pattern for the first IMF, which calls for an additional layer of the HHSA, and an additional dimension. The Metonic cycle is now clearly shown in the sixth IMF. The axis labels are the same as in figure 9*b*, with the number of data points making up the *x*-axis, and the *y*-axis representing arbitrary units. (*c*) The Hilbert time–frequency spectrum from the IMFs given in figure 9*b*.

**Figure 10.**
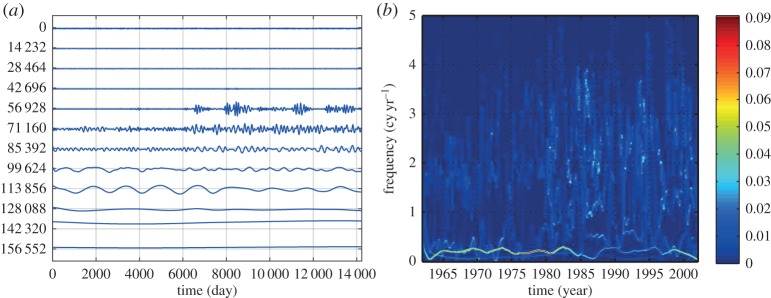
(*a*) The second layer of HHSA for the LOD data. Here is the IMFs for the first component in figure 9*b*. The Olympiad cycle (4 years) is now clearly shown in IMFs 8 and 9. The axis labels are the same as in figure 9*b*. (*b*) The Hilbert time–frequency spectrum confirms the Olympiad cycle, which could be included in a higher-dimensional HHS.

**Figure 11.**
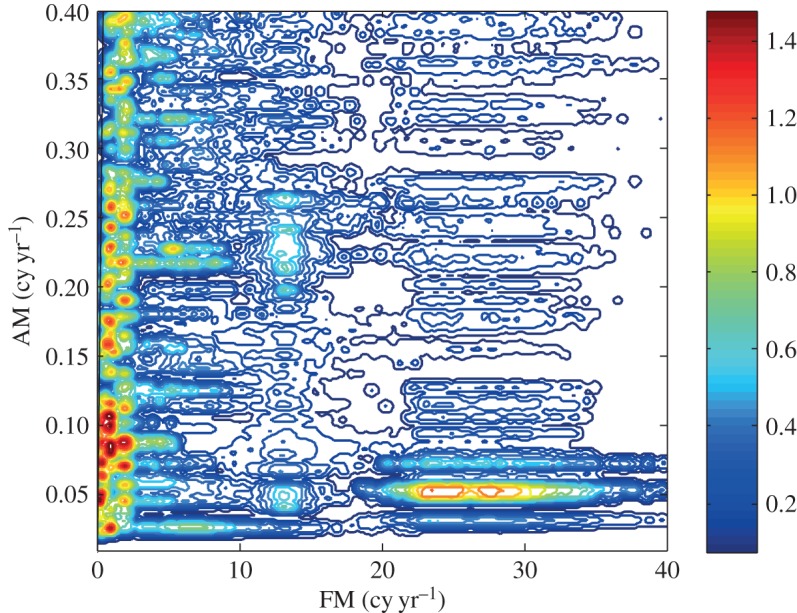
The HHS for the LOD data. Here we can see the modulating patterns all in one figure, representing the cross-scale nonlinear interactions. Again, the *x*-axis is the carrier frequency and the *y*-axis is the AM frequency. The half-monthly tide (having a frequency with intra-wave modulation) covers the range from 20 to 35 cycles per year, and is modulated by the 19-year Metonic cycle modulation. The richest energy distribution is on the annual cycle modulated by motions of many different scales, which represent the complicated nonlinear ocean dynamic processes.

HHSA could be applied to all kinds of time-series data. It is especially useful for data from complex systems where inter-mode interactions are the prevailing processes. We will demonstrate the power of HHSA in the following example for a geophysical dataset of the length of day (LOD), which has been studied extensively in [13]. From the data in figure 8*a*, we can see some very large-baseline fluctuations, which must be an additive process, i.e. a lower-frequency oscillation carries a higher-frequency one spread evenly above and below the carrier. The IMFs produced by EMD are given in figure 8*b* and the Hilbert time–frequency spectrum in figure 8*c*. One of the most prominent features of the IMFs is the 19-year Metonic cycle-produced AM. It is seen in the component representing the half-monthly tide, but it is not in any of the IMFs. It should be noted that the large amplitude in the third from the bottom IMF is not the Metonic cycle, for it has the wrong period, phase and magnitude. No amount of filtering and decomposition could extract this feature in a spectral representation. The Hilbert spectrum in the time–frequency form does show some energy fluctuation at 19- and 4-year cycles, shown only in the incomplete time–frequency representation. However, the marginal spectrum, as given in [13], for example, would average out the energy fluctuation and only show the peaks at half-monthly, monthly, half-yearly and yearly frequencies. Yet, it could be clearly seen if we construct the envelope of the half-monthly tidal component, as shown partly in figure 9*a*. An EMD of this envelope produces the second layer IMFs as given in figure 9*b*. In this set of IMFs, we can see the Metonic cycle clearly in the sixth IMF component (sixth from the top). Interestingly, there are still strong AMs in the first component. This modulation could be seen also in the Hilbert time–frequency spectrum in figure 9*c*, which gives a roughly 4-year cycle. The existence of the strong AM calls for an additional layer of analysis. The EMD analysis actually reveals a strong wave pattern of the 4-year Olympiad cycle as in figure 10*a*, and the corresponding Hilbert time–frequency spectral representation in figure 10*b*. Thus, we finally recovered all the possible patterns in the data through three layers of HHSA. Both the Olympiad and Metonic cycles have their rightful astronomical significance, respectively. (The 19-year Metonic cycle is remarkable for being nearly a common multiplier of the solar year and the lunar month.) Yet, there is no filtering technique that could reveal this hidden scale in the data so readily as in HHSA. The three-dimensional HHS is given in figure 11. Here we can see the modulation patterns for all the different time scales including the Metonic (1/19 is approx. 0.052) and Olympiad cycles, which are totally absent in the time–frequency Hilbert spectrum representation in figure 8*c*. This example demonstrates that a fully informational high-dimensional spectrum could represent all cycles without resorting to the time–frequency representation, which, in our opinion, is an incomplete spectral representation.

**Figure 12.**
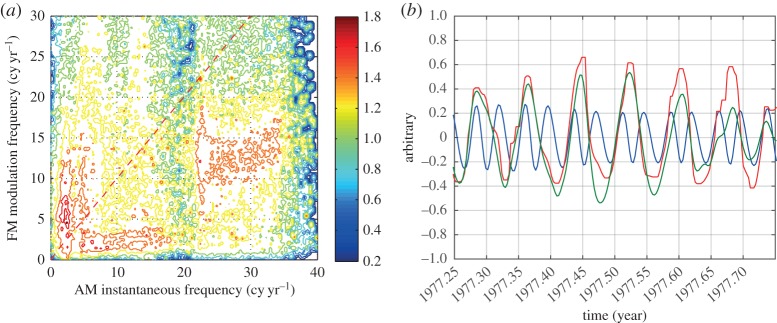
(*a*) The AM–FM spectrum of LOD. The *x*-axis is the carrier frequency; the *y*-axis is the FM. Here the emphasis is on the FM of the LOD data up to the annual cycles. For the first time, we find that the frequency of the half-monthly tide is actually modulated by the monthly cycle. Because the FM could be both intra- and inter-wave, this should be a full-plane representation. The red dotted line divides the plane into inter-wave (lower half) and intra-wave (upper half) modulations. (*b*) The verification of the FMs (red solid line) by the temporal domain data: the frequency variation of the half-monthly tide (blue solid line) is modulated by a monthly cycle (green thin solid line).

Next, we will examine the AM–FM spectrum to represent some similar but slightly different phenomenon: the AM–FM interactions. Let us examine the first IMF of the data, which represents the half-monthly component in figure 12*a*. The modulation relationship between the AM frequency (half-monthly data) and its FM instantaneous frequency is clear now: the half-monthly AM frequency is actually modulated by the monthly FM frequency, which could also be seen in the data as in figure 12*b*, where the vertical scale is adjusted for visual comparison. Here the monthly AM cycle is modulated by a half-yearly FM cycle (2 cycles per year). This various cross-scale modulation is also absent in the time–frequency Hilbert spectrum in figure 8*c*. It should be pointed out that, unlike the Holo-Hilbert AM spectrum, the AM–FM spectrum is a full-plane presentation divided into two halves by the dotted line representing a modulating frequency equal to the frequency. The half plane above the line represents intra-wave FM and the half plane below, the inter-wave modulation. The FM modulation could be larger than, equal to, or smaller than the AM frequency depending on the system characteristics as shown in the intra- or inter-wave degree of nonlinearity. For example, we can see that the FM modulation for the AM instantaneous frequency near the 2 cycles per year value is near 6 cycles per year. Therefore, the ratio is 3 to 1, implying that the oscillation is intra-mode nonlinear to the fourth order rather than being the subharmonic in the half-monthly oscillations. This also illustrates the amount of information produced by this new approach.

**Figure 13.**
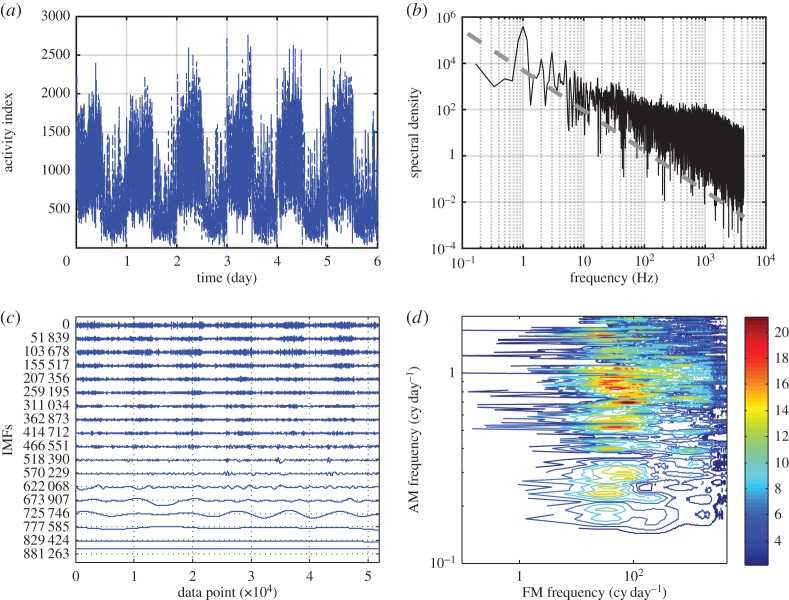
(*a*) The neuron population activity index data collected *in vivo*, from the suprachiasmatic nucleus (SCN) region of a laboratory mouse. A circadian cycle (24 h) is clearly seen. (*b*) The Fourier spectrum of the neuron population activity index data shows a power-law tail implying simple self-similarity. The grey dashed line is a −5/3 power law, with spectral density in terms of the activity index (vertical scale). (*c*) The IMFs of the neuron population activity index data: though the circadian daily cycle is visible, the strong modulation patterns in the first 12 components are also clearly visible and in step with the circadian cycles of components 14 and 15, indicating nonlinear cross-scale interactions. The vertical scale is based on the activity index. (*d*) The HHS of the neuron population activities data: here the strong circadian modulation (1 cycle per day or 24 h period) in amplitude (*y*-axis) is clearly seen for neuron activity frequency on the *x*-axis covering a wide range from 10 to 1000 Hz.

To demonstrate the usefulness of the HHSA, we will now consider the case of neuron population activities. The data are collected *in vivo*, from the suprachiasmatic nucleus (SCN) region of a laboratory mouse, where the neurons are endowed with the clock gene, called the *zeitgeber*. The population activity index data, shown in figure 13*a*, represent the sum of total neurons firing, each at a different rate, in a given time interval of 10 s. A Fourier spectral representation is given in figure 13*b*, which shows a prominent circadian cycle peak at 1 cycle per day, and a long power-law tail, a typical noise-like signal, which is usually interpreted as pure noise with no structure other than self-similarity, a phenomena almost exactly like the wave–turbulence interaction processes discussed by [9]. Indeed, the neuron population data have been interpreted as such. If we perform an EMD on the data, we would obtain 18 IMF components shown in figure 13*c*. Here the circadian cycle is clearly seen in components number 14 and 15. There are, however, AMs at the circadian cycle in all the higher-frequency components in IMF numbers 1–10. Unfortunately, neither the Fourier nor the Hilbert spectrum would show these multiplicative modulations. The HHSA of the first 10 IMFs from the data yields the HHS as given in figure 13*d*. Those components are all of higher frequency than the circadian cycle: from the frequency axis, we can see the energy showing prominent circadian cycle modulation is residing in the range covering from 10 to 2000 Hz, an indication of the nonlinear multiplicative modulation of the neural activity index by the circadian cycle. Furthermore, the higher-energy components can also be shown to phase-lock to the circadian cycle as was shown in the wave–turbulence interaction cases [9]. These and many other properties will be discussed in other papers in this issue. It suffices here to say that the HHSA can indeed reveal detailed nonlinear processes inaccessible to other methods.

## 5. The time-domain amplitude modulation analysis

The AM could be studied in both the time and frequency domains. Let us now discuss the amplitude fluctuations in the time domain. Knowing the importance of the amplitude functions, it is surprising to see that, up to this time, the study of AM fluctuations in data has never attracted as much attention as the FM. One of the difficulties in amplitude-related analysis is how to define the amplitude fluctuation for a non-stationary and nonlinear signal in general. Ideally, the amplitude should be the envelope to a FM carrier. A well-known tool, the Hilbert transform, seems to fit our need. But as discussed by Huang *et al.* [2,11], the Hilbert transform could not extract a well-behaved smooth envelope for an arbitrary signal. Furthermore, the Bedrosian theorem [14] clearly indicated that the Hilbert transform would favour the fast-changing component of the signal. In the case of an AM signal, the Hilbert transform would only compute frequency from the higher carrier wave components. In most cases, the Hilbert transform-defined envelopes are also highly oscillatory and verge on nonsensical results [3]. In fact, the envelope for an arbitrary time series cannot be defined meaningfully as discussed in [11]. The only way a unique envelope can be defined is through the IMF [2]. This requires the time series to be decomposed by the EMD first, as given in equation (1.3).

For any process having prominent cyclic variations, the time-domain analysis is especially effective. Let us designate all the prominent modes with discernible modulation patterns as waves and the rest as random modulations with no features. Then for any additively decomposed component, we will have
5.1
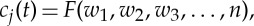
with *w*_*h*_ as waves and *n* as featureless noise. It is possible to represent an amplitude function as the sum of waves and noise both additively and multiplicatively. Indeed, any prominent pattern can be used to demodulate either additively or multiplicatively until the residue is rendered as very noise-like. Equation (1.3) dictates that an additive demodulation can be performed easily on the IMFs:
5.2
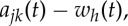
with *w*_*h*_(*t*) as the prominent mode of the IMF in equation (1.3). An additive demodulation would move the local mean of the data to the zero axis. After the additive demodulation, if there is any AM pattern, *w*_*r*_(*t*) say, in the residue, we can demodulate the residue with this specific wave pattern multiplicatively (not necessarily of the same pattern as *w*_*h*_(*t*)) to extract a second layer residue from the corresponding IMFs. This process could be iterated as
5.3
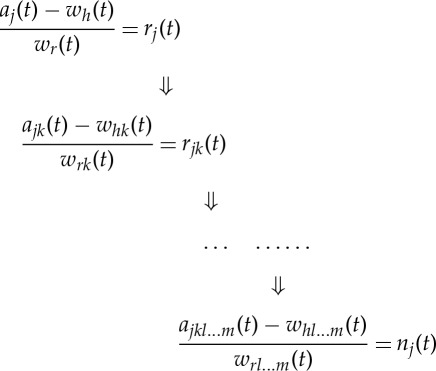
so that at each iteration, we will have
5.4
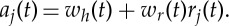
Eventually, the data should be expressed as a collection of oscillations and noise with no discernible patterns. It is possible that the data can be all positive with prominent AMs. In this case, the multiplicative demodulation should be implemented. The power of this time-domain analysis is illustrated clearly by the study of wave–turbulence interactions given by [9], also in this theme issue. In their study, they have employed the time-domain analysis to separate wave and turbulence, which enabled them to conduct a detailed study of the interaction processes.

This is thus the object of time-domain analysis: *to delineate the linear additive and nonlinear multiplicative inter-mode interactions*. The above time-domain view is very useful when the modulating patterns are cyclic and simple, such as the circadian phenomena. If the modulating patterns are complicated, we have to resort to the full HHSA that would reveal patterns involved in all the possible additive or multiplicative interactions. A useful index to quantify the role of any prominent cyclic terms is to define an inter-mode linearity index,
5.5
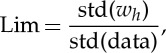
as a measure of the energy in the additive wave pattern component. The higher this value is, the more linear is the inter-mode interaction. Of course, the constituent components could be either intra-mode linear or nonlinear.

## 6. Summary discussion and conclusion

Throughout the above discussions, we have emphasized the difference between linear additive and nonlinear multiplicative interactions. An interesting point here is that in Fourier analysis, for simple trigonometric terms, there is an equivalence between additive and multiplicative operations as shown in equations (2.3) and (2.4). Therefore, we have an ambiguity, which has to be resolved in order to determine which case is indeed additive but not multiplicative. The basis in all our expansions is the adaptive IMFs in the form of
6.1
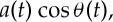
in which *a*(*t*) is the amplitude of the unit phase function cos *θ*(*t*). This form could have a wide band representation in Fourier analysis. The advantage is that the form might have genuine physical meanings as demonstrated by [2]. Furthermore, the modulations of *a*(*t*) and cos *θ*(*t*) represent inter- and intra-mode nonlinearities. Together, they could represent a complicated multiplicative signal in one wave component. Of course, not all additive terms are equivalent. Depending on the amplitude and frequency ratios of the terms involved, [15] made a detailed study. They concluded that ’EMD allows one to address in a fully data-driven way the question whether a given signal is better represented as a sum of two separate, un-modulated tones, or rather as a single, modulated waveform, with an answer that turns out to be in good agreement with intuition (and/or perception).’ We like to use the sparsity principle here as an additional guide and criterion, which stipulates that the correct wave component decomposition is the one with the least number of constituting components. For example, a product term is one component, but the equivalent two-component sum calls for two terms; therefore, the correct choice with this criterion to best represent the data is the one-term IMF.

Another example is
6.2
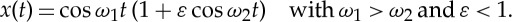
This expression is a single-component IMF in HHSA; it represents a carrier at frequency *ω*_1_, and a modulation amplitude *ω*_2_. In the Fourier representation, however, it would have to have three terms at *ω*_1_, *ω*_1_+*ω*_2_ and *ω*_1_−*ω*_2_ for the central frequency and sidebands. We believe IMF always represents a more physically meaningful wave; therefore, the less the number of components, then the closer to the truth physically. When we have a more complicated case than just the two-term situation as in the sinusoidal and white noise pairs discussed above, the situation is much clearer on the nature of multiplicative interactions.

Now we consider the quantification of the inter-mode coupling and the degree of nonlinearity. In our discussion of the inter-mode nonlinearity above, we have proposed a simple one-number linearity index given in equation (5.1) for the case when a prominent additive cycle is present. For the general cases, a more refined and useful index could also be introduced to quantify the detailed distribution of the nonlinearity in the AM–FM frequency space through the area coverage of the HHS.

Another more direct way to quantify nonlinearity is to index the inter-mode degree of linearity or nonlinearity. As discussed above, the linearity should be expressed in terms of the additive decomposition components, while the nonlinearity should be expressed in terms of multiplication-induced AM. In the EMD expansion of the amplitude given in equation (1.3), if we take the sum without the last trend term, if any, as
6.3
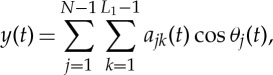
then every term is zero-mean and orthogonal to each other. Consequently, the total energy of the AM should be
6.4
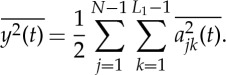
As a result, we can define the degree of inter-mode nonlinearity, the depth of the modulation, as
6.5
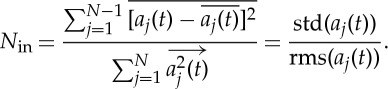
Based on this definition, the maximum nonlinearity should be unity, when the trend term for the amplitude function is identically zero, then the total energy of the amplitude is identical to that of the signal. This is total modulation; the degree of nonlinearity should be unity, the highest value possible. In all other cases, the value should be less than unity. The other extreme is for all the amplitudes to be constant while the modulation is zero, or a linear case. Thus we should have a degree of inter-mode nonlinearity between 0 and 1, just as in the case of the intra-wave degree of nonlinearity.

Through a detailed study of the dynamics, we have established that there are two basic interaction processes: the additive and the multiplicative. The former is linear, while the latter is nonlinear. In the complicated fluid dynamics setting, the kinematics is controlled by linear processes, while the dynamics is controlled by nonlinear processes. Although both additive and multiplicative processes could generate AMs, the linear additive processes could be successfully treated by the existing additive decompositions such as Fourier, wavelet and Hilbert–Huang transform (HHT). The nonlinear multiplicative cases present a problem.

What we propose here is to augment the capability of the existing spectral analysis by introducing additional dimensions to extract information from the nonlinear multiplicative processes. All existing spectral analysis methods, be it Fourier, wavelet, Wigner–Ville and HHT, suffer from the limitation imposed by the additive basis expansions, *a priori* or *a posteriori* (adaptive). Thus the information the method reveals is limited to linear processes. HHT based on the nonlinear EMD did expand the HSA into intra-mode nonlinearity, but EMD is still a formal additive expansion. Therefore, it would not be able to study the phenomena involved in nonlinear inter-scale coupling fully. With the new HHSA we can effectively study both intra- and inter-mode nonlinearity. Some clarifications are needed here concerning the inter- and intra-mode nonlinearities.

The EMD is a formal additive decomposition method. Theoretical and logical arguments would dictate that each IMF mode from EMD should have some dynamical significance, for only the significant dynamics could generate a signal strong enough to present local extrema. The underlying dynamical mechanism (i.e. control equations) could make the corresponding IMF to be intra-mode nonlinear, as discussed by [4,16]. Additionally, the interactions among the different IMFs could also be either linearly additive or nonlinearly multiplicative. It should be pointed out that the IMF components involved in nonlinear inter-mode interactions might not be nonlinear themselves, but any multiplicative process would produce effects undetectable by any of the existing spectral methods based solely on additive decomposition methods. Therefore, we should beware the nonlinear interactions that could be mistaken for linear processes. The quantification of the total degree of nonlinearity, as a sum of the intra- and inter-mode degree of nonlinearities, could have many applications. For example, it could be used for machinery health monitoring. If the vibration is smooth and linear, it should be in good operational condition. On the other hand, if the vibration signal is full of AM, then the vibration is the consequence of nonlinear processes, which could lead to instability and machine damage. The machinery should then be carefully inspected.

The difference between the present HHSA and the other spectral analysis methods can be summarized as in table 1.

**Table 1.** Comparison of spectral analysis methods. F, frequency; TF, time–frequency

	decomposition			nonlinearity	
methods	additive	multiplicative	non-stationary	intra-mode	inter-mode
Fourier	yes	no	no	maybe?	no
Hilbert: TF	yes	no	yes	yes	no
Hilbert: F	yes	no	no	yes	no
HHSA: TF	yes	yes	yes	yes	yes
HHSA: F	yes	yes	yes^a^	Yes	yes

^a^HHSA in frequency space could represent non-stationary processes, for we can add more dimensions to exhaust all the AMs.

The newly proposed HHSA method (based on EMD analysis) would systematically delineate, clarify and quantify the effects of linear and nonlinear intra- and inter-mode interactions. Thus it liberates the spectral analysis from the present limitations imposed by Fourier, wavelet and even HHT. Obvious applications are numerous: for example, the predicament and lack of a breakthrough on EEG-based brain studies would be resolved and revolutionized with the clarification and quantification of inter-mode interactions. These results will be reported separately in other papers in this issue. The HHSA could also resolve the detailed dynamics in turbulent flow [9]. With HHSA, we finally get a full spectral representation for nonlinear and non-stationary data, with all the possible modes of AM and FM, both additive and multiplicative, opening a new and unique view of the data filling the world around and in us all.

## Data accessibility

The LOD from the comb2011_noon.eop series is available at ftp://euler.jpl.nasa.gov/keof/combinations/2011.
